# Analysis of factors influencing the use of child restraint system by parents of children aged 0–6 years: an information, motivation, behavioral skills model-based cross-sectional study

**DOI:** 10.1186/s12887-022-03827-9

**Published:** 2023-01-02

**Authors:** Yaru Sun, Ting Liu, Junyu Chen, Juan Huang, Xin Wang, Mingqin Lu, Ying Luo, Xiuling Yang

**Affiliations:** 1 School of Nursing, Qingdao University, No.38 Dengzhou Road, Qingdao, 266021 Shandong Province China; 2 The Affiliated Hospital of Qingdao University, Qingdao, Shandong Province China

**Keywords:** Parents, Child restraint System, IMB model, Influencing factors

## Abstract

**Background:**

Children's injuries from traffic accidents have been identified as a global public health issue. Child restraint system (CRS) is a useful tool for lowering the risk of injury to children. Nevertheless, CRS usage is really low in China. The goal of the current study was to investigate the use of CRS after the legislation revised in China and to explore the influencing factors based on Information, Motivation, and Behavioral Skills model (IMB).

**Methods:**

The study is a cross-sectional survey of parents who took their 0 to 6-year-old children for seeking primary care services at the Children Preventive Health Care Clinic of a tertiary hospital in Shandong Province, China. Parents were invited to complete the self-administered questionnaire between March and June 2022, including their knowledge, motivation, and behavioral skills, use behavior of CRS and socio-demographics. Ordinal logistic regression was used to explore the factors associated with CRS use by using SPSS software (version 26.0).

**Results:**

In total, 442 parents participated in the study; 56.1% (*n* = 201) of the parents utilized CRS for their child passengers, however only 29.0% used CRS frequently. The result of logistic regression analysis show that parents with junior college (OR = 0.398, 95%CI: 0.185 ~ 0.857), possessing a high family economic status(OR = 0.225, 95%CI: 0.088 ~ 0.578), being trained on children’s unintentional injuries(OR = 0.435,95%CI: 0.272 ~ 0.695), and having high scores on CRS riding mode cognition(OR = 0.476, 95%CI: 0.368 ~ 0.616), CRS type cognition(OR = 0.519, 95%CI: 0.392 ~ 0.689), CRS use motivation(OR = 0.392, 95%CI: 0.295 ~ 0.520) and installation skills(OR = 0.559, 95%CI:0.411 ~ 0.761) were the main factors promoting the usage of CRS.

**Conclusions:**

This study found that the use of CRS can be increased by improving parents' knowledge, motivation and behavior skills and hence related educational programs is necessary for increasing CRS use in China.

**Supplementary Information:**

The online version contains supplementary material available at 10.1186/s12887-022-03827-9.

## Background

In recent years, private automobiles have displaced public transportation as children's principal source of transportation [1]. The ever-increasing complexity of the traffic situation on the roadways posed a grave threat to the safety of children [2]. Approximately 10,000 children die annually from traffic-related injuries on Chinese roadways [3], with children aged 0 to 6 years old accounting for 60% of these deaths and riding accounting for 44% of all fatalities [4]. Over the past 10 years, child fatalities without use of the CRS have accounted for a higher proportion of child traffic injury deaths, averaging 43.9% [5]. In addition, studies have shown that infants and children who did not use CRS in vehicles are three times more likely to be injured and up to eight times more likely to die [6]. Due to parental neglect or inappropriate usage of Child Restraint Systems (CRS), the number of children injured while riding in automobiles continued to increase [7].

CRS has the potential to significantly lower infant mortality rates by more than 60% [8]. Most countries in the EU have legislation mandating the use of safety restraints for children traveling in cars, and CRS use rates in these countries have generally reached 90% after the legislation [9], and countries have seen significant decreases in child passenger injuries, such as the United States and Germany, where the number of child road traffic deaths has decreased by about 50%, only 76 children died in cars in Germany in 2011 [10]. However, less than 20% of CRS utilization occurred in China, which was significantly lower than the 90% utilization rate in other countries [11, 12]. In an effort to boost the utilization rate of CRS in China, the newly revised "Law on the Protection of Minors" includes CRS for the first time in national legislation in June 2022 [13]. In addition, the "Guidelines for Development of Chinese Children (2021–2030)" issued by the State Council mandated that CRS will be promoted and implemented in September 2022 [14]. However, CRS legislation has just been put into effect, and no appropriate sanctions have been put in place to enforce its usage. The legislation has to be further improved and publicized. Under the background of the introduction of legislation, the strategies of improving the use of CRS should be explored. Nevertheless, there were few studies on the factors affecting the use of CRS in China [15].

**Fig. 1:**
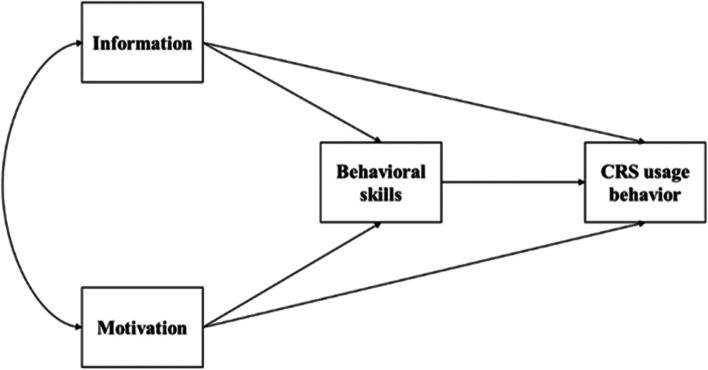
Information motivation behavior skill model of CRS usage behavior

Information, Motivation, and Behavioral Skills model (IMB), one of the widely used theories for comprehending and assessing health behaviors, contained three components: information, motivation, and behavior skills [16]. Personal and social motivation includes attitude and social support, respectively. Behavioral skills consist of personal objective skills for performing the behavior and a perceived sense of self-efficacy [17]. This model posits that information and motivation influence behaviors directly or indirectly while behavioral skills directly influence behaviors [18]. The usefulness of IMB model has been proven continuously in various fields of medical care [19–21]. Figure 1 shows the IMB model framework used in this study.

Existing research on factors influencing parental behavior in CRS use has focused on demographic base information and parental theoretical perceptions, and there is a lack of research parents' motivation and objective skills of using CRS [22]. Research has shown that incorrectly installed CRS increases the risk of child injury and death in accidents. Since the installation of CRS is mostly done by parents and the installation is difficult, it is a very challenging job for parents. Due to the lack of installation skills, CRS installation errors often occur, which poses a huge threat to children's safety. So it is equally important to understand the objective skills of parents for CRS use [23]. In order to offer a scientific basis for further promoting the use of CRS in China, this study aimed to examine, using the IMB model, the factors that influence parents' usage of CRS for children aged 0 to 6 years.

## Methods

### Sample size calculation

The sample size was determined by using a single population proportion formula with a 95% confidence level and 5% margin error. The actual sample size for the study was computed by using $N=\frac{{{Z}^{2}}_{1-\frac{\alpha }{2 }} p(1-p)}{{\delta }^{2}}$, where α = 0.05, Z_1-α/2_ = 1.96, δ = 0.05, Based on a prevalence of 17.3% [11], including the 20% non-response rate, the minimum sample size was calculated as 264. Finally, 460 questionnaires were distributed in total.

### Participant recruitment

The present study is a cross-sectional study with approval by the medical ethics review board (QDU-HEC-2021176). The study was conducted in the Children Preventive Health Care Clinic of a tertiary hospital in Shandong Province, China. Parents who took their children to seek primary care at the Clinic between March and June 2022 were included in the study. All eligible participants who visited the clinic during recruitment were invited to participate in the survey. Inclusion criteria were: (1) Father or mother of children aged 0–6; (2) parents aged ≥ 18 years; and (3) parents owning one or more private vehicles [(Sedan, Sport utility vehicle (SUV), etc.]. Exclusion criteria of parents were: (1) refuse to participate in the research; (2) do not drive a private car to pick up the child; and (3) severe mental illness or cognitive impairment.

### Data gathering procedure

Questionnaires were collected by trained investigators in the form of online and offline interviews. The wjx.cn platform was used to distribute the online surveys, and face-to-face interviews with parents were conducted by researchers to collect data offline by using the same questionnaires. The investigator thoroughly explained the contents and precautions of the questionnaire before it was filled out, and asked the respondent to complete any missing information and correct illogical questions on spot. All participants sign the informed consent form. The investigator selected 5% of the completed questionnaires at random for a second inspection, and if there were no errors, two individuals entered the data into the electronic database.

### Data collection tools

#### General information questionnaire

A general informational questionnaire was designed using expert consultation and a review of the literature. There were two sections in the questionnaire: (1) Demographic data on parents' highest level of education, income level, child's gender, age, registered address, home ownership, and unintentional injury training participation experience; and (2) CRS usage status; High frequency use refers to CRS being used over 5 times in an average of 10 rides, low frequency use is defined as CRS being used between 1—5 times, and no use indicates that CRS is not used. [Additional file 1].

#### IMB model questionnaire

The questionnaire was developed through a literature review, consultation with specialists and in-depth interviews with parents of children aged 0–6. During the literature review, we mainly referred to articles by previous research [24, 25]. There are 5 dimensions and 18 items in the IMB questionnaire. The knowledge related to CRS use consisted of 7 items and were divided into riding mode cognition dimension (internal consistency α = 0.902, 4 items) and type cognition dimension (internal consistency α = 0.789, 3 items), assesses the participants' cognition on the age of CRS use, child seating position and seat type replacement. The motivation dimension (internal consistency α = 0.789, 5 items) assesses participants' personal motivation and authoritative advice on CRS use. The behavioral skills related to CRS use consisted of 8 items and were divided into self-efficacy dimension (internal consistency α = 0.808, 3 items) and installation skills dimension (internal consistency α = 0.756, 3 items). The questionnaire adopts Likert’s 5-point scoring, with 1–5 representing from “Strongly Disagree” to “Completely Agree”. The study examined the reliability of 18-item questionnaire, and indicated that the Cronbach's α for the entire questionnaire was 0.887. Additionally, the study used exploratory variables to confirm the questionnaire's structural validity, with KMO = 0.828 and the Bartlett spherical test value of 2180.8 (*P* < 0.001). [Additional file 2].

### Data analysis

Statistical Package for Social Sciences (SPSS) version 26.0 was used for all statistical analyses. The normal distribution of continuous variables was assessed by kurtosis and skewness. Means and standard deviation were used to describe measurable data that follow the normal distribution. Categorical variables were described as frequency and percentage, and Chi-square test was used for comparison. One-way analysis of variance (ANOVA) was used to compare the mean scores among three or more groups for continuous variables. Ordinal logistic regression analysis was used to identify the variables affecting parents' use of CRS, with the odds ratios (OR) and their corresponding 95% confidence intervals (CI) presented. Statistical significance was established when *P* < 0.05.

## Results

### Participant characteristics

There were 460 questionnaires distributed in total, and 442 valid questionnaires were returned, with a response rate of 96.1%. Among them, 220 valid questionnaires were collected online and 222 offline. Table 1 displays the demographic details of the parents and their children. In the current study, the majority of respondents was mothers (70.4%); 66.7% of respondents had a college degree or higher. The majority of respondents (77.8%) were registered residents of local city households. Children aged 0–3 accounted for 44.3%, and children aged 4–6 accounted for 55.7%, though, reported they had not participated in the training on child accident injuries. 56.1% of parents used CRS for their children, however only 29.0% of parents Frequently used it. The CRS usage of respondents in different characteristic groups was compared, and the results showed that parental role, educational background, monthly income per capita, family status, house ownership and unintentional injury training participation experience were statistically associated with CRS use(*P* < 0.05). (Table 1).

**Table 1:** CRS usage of respondents grouped by different characteristics

Variable	Category	Number of no usage(Proportion)	Number of low frequency usage(Proportion)	Number of high frequency usage(Proportion)	χ^2^	*P*-value
Respondents	Mother	148(47.6%)	77(24.8%)	86(27.7%)	6.095	0.047
	Father	46(35.1%)	43(32.8%)	42(32.1%)		
Education	Bachelor degree or above	45(30.0%)	57(38.0%)	48(32.0%)	56.703	**< **0.001
	Junior college	50(34.5%)	43(29.7%)	52(35.9%)		
	High school, middle special or technical school	46(59.7%)	12(15.6%)	19(24.7%)		
	Middle school or lower	53(75.7%)	8(11.4%)	9(12.9%)		
Household per capita monthly income (yuan)	≤ 3000	44(80.0%)	5(9.1%)	6(10.9%)	62.860	**< **0.001
	3001–6000	92(49.7%)	44(23.8%)	49(26.5%)		
	6001–9000	42(35.3%)	45(37.8%)	32(26.9%)		
	> 9000	16(19.3%)	26(31.3%)	41(49.4%)		
Family status	Only child	68(37.4%)	52(28.6%)	62(34.1%)	6.021	0.049
	Non-only child	126(48.5%)	68(26.2%)	66(25.4%)		
Gender	Male	105(41.8%)	70(27.9%)	76(30.3%)	1.027	0.598
	Female	89(46.6%)	50(26.2%)	52(27.2%)		
Age group (years)	0–3	78(39.8%)	63(32.1%)	55(28.1%)	4.678	0.096
	4–6	96(47.2%)	50(23.2%)	43(29.7%)		
Household registration	Qingdao	148(43.0%)	94(27.3%)	102(29.7%)	0.541	0.763
	Non-Qingdao	46(46.9%)	26(26.5%)	26(26.5%)		
Housing ownership	Personally owned	156(40.7%)	104(27.2%)	123(32.1%)	16.395	**< **0.001
	Rent	38(64.4%)	16(27.1%)	5(8.5%)		
Unintentional injury training participation experience	Yes	41(30.8%)	33(24.8%)	59(44.4%)	23.364	**< **0.001

### IMB questionnaire score

In the group with the highest frequency of usage, the scores of IMB dimensions ranked from high to low as follows: CRS use motivation (4.14 ± 0.74), self-efficacy (4.06 ± 0.68), riding mode cognition (3.96 ± 0.87), installation skills (3.73 ± 0.75) and type cognition (3.39 ± 0.80). Except for the use motivation score, which was lower in the high frequency use group than in the low frequency use group, the scores of all other dimensions were higher in the high frequency use group than both the low frequency use group and the non-use group (*P* < 0.001). The CRS use motivation scores differed significantly from one another (*F* = 129.739, *P* < 0.001), which is shown in Table 2.

**Table 2:** IMB dimension scores of different CRS usage behaviors

Dimension	Average	CRS usage behaviors			*F*	*P*-value
		**Non-use group**	**Low frequency use group**	**High frequency use group**		
Riding mode cognition	3.39 ± 1.07	2.73 ± 1.01	3.86 ± 0.69	3.96 ± 0.87	95.787	**< 0.001**
Type cognition	3.00 ± 0.90	2.52 ± 0.88	3.35 ± 0.65	3.39 ± 0.80	60.383	**< 0.001**
CRS use motivation	3.58 ± 1.08	2.84 ± 1.08	4.18 ± 0.47	4.14 ± 0.74	129.739	**< 0.001**
Self-efficacy	3.81 ± 0.64	3.64 ± 0.51	3.83 ± 0.69	4.06 ± 0.68	18.089	**< 0.001**
Installation skills	3.34 ± 0.92	2.88 ± 0.95	3.67 ± 0.68	3.73 ± 0.75	54.380	**< 0.001**

### Analysis of influencing factors of usage behavior of CRS

With independent variables confirmed as being statistically associated with the use of CRS, multiplicity tests should be done for the significance. The results show that there is no multicollinearity between variables (VIF < 10) (Table 3). Then, the research has passed the parallel line test, ordinal analysis was conducted. Table 4 demonstrates the impact of several variables on parental use of CRS. The respondents with junior college education, possessing a high family economic status, being trained on children’s accidental injuries, and scored highly on riding mode cognition, type cognition, CRS use motivation, and installation skills were the factors promoting the use of CRS.

**Table 3:** Test for multicollinearity between variables

**Variable**		**VIF**
Respondents	Mother	1.067
	Father	
Family status	Non-only child	1.067
	Only child	
Education	Middle school or lower	1.206
	High school, middle special or technical school	
	Junior college	
	Bachelor degree or above	
Household per capita monthly income (yuan)	≤ 3000	1.160
	3001–6000	
	6001–9000	
	> 9000	
Housing ownership	Rent	1.063
	Personally owned	
Children's unintentional injury training participation experience	No	1.039
	Yes	
Riding mode cognition		1.390
Type cognition		1.231
CRS use motivation		1.470
Self-efficacy		1.134
Installation skills		1.284

**Table 4:** Logistic regression analysis of factors for frequency of CRS usage

Variable	B	SE	Waldχ2	*P*-value	OR (95%CL)
Respondents					
Mother					1 (ref.)
Father	-0.254	0.241	1.116	0.291	0.775(0.484 ~ 1.423)
Family status					
Only child					1 (ref.)
Non-only child	0.413	0.229	3.258	0.071	1.511 (0.965 ~ 2.368)
Education					
Middle school or lower					1 (ref.)
High school, middle special or technical school	-0.504	0.43	1.371	0.242	0.604(0.260 ~ 1.404)
Junior college	-0.921	0.391	5.549	0.018	0.398(0.185 ~ 0.857)
Bachelor degree or above	-0.352	0.397	0.788	0.375	0.703(0.323 ~ 1.530)
Household per capita monthly income (yuan)					
≤ 3000					1 (ref.)
3001–6000	-0.576	0.444	1.685	0.194	0.562(0.235 ~ 1.342)
6001–9000	-0.619	0.459	1.816	0.178	0.539(0.219 ~ 1.325)
> 9000	-1.491	0.481	9.596	0.002	0.225(0.088 ~ 0.578)
Housing ownership					
Rent					1 (ref.)
Personally owned	-0.755	0.369	4.196	0.041	0.470(0.228 ~ 0.968)
Children's unintentional injury training participation experience					
No					1 (ref.)
Yes	-0.883	0.239	12.114	0.001	0.435(0.272 ~ 0.695)
Riding mode cognition	-0.742	0.131	32.051	**< **0.001	0.476(0.368 ~ 0.616)
Type cognition	-0.655	0.144	20.696	**< **0.001	0.519(0.392 ~ 0.689)
CRS use motivation	-0.937	0.145	42.012	**< **0.001	0.392(0.295 ~ 0.520)
Self-efficacy	-0.149	0.181	0.675	0.411	0.862(0.604 ~ 1.229)
Installation skills	-0.581	0.157	13.686	**< **0.001	0.559(0.411 ~ 0.761)

## Discussion

In the present study, the CRS utilization rate was 56.1%, and the high frequency use rate was 29.0%, both of which are higher than the CRS utilization rate in the country as a whole and in other regions in China [26–28]. This may be a result of the state's increased promotion of the benefits of CRS use in recent years [29]. However, the rate is still quite inferior to that in other countries [30]. The primary cause is the early implementation of required measures in other countries to penalize parents who did not use of CRS [31]. This finding acted as a reminder to China's relevant ministries to increase the law's universality and enforceability. Local governments and organizations also need to execute targeted penalties expeditiously to increase the CRS utilization rate.

According to research, families with strong financial standing are more likely to use CRS, which is in line with the findings of previous studies [32]. In order to address the low purchase rate caused by the price, foreign nations provided complimentary safety baskets for infants to assure their safety on the journey home [33]. In China, it is difficult to freely offer CRS to every household with children and a car due to economic concerns [34]. However, by offering discount coupons or low-cost rentals, the pertinent departments can encourage parents to buy and use CRS [35]. Furthermore, parents who have attended training on unintentional child injuries had a high rate of CRS utilization. Because those parents had more opportunities to learn about CRS knowledge [36], which also signifies that they would have greater capacity to prevent riding-related accidents in injuries to children if they possessed a greater amount and reliability of knowledge [37]. The results of the univariate analysis of this study indicated that family status was associated with CRS use. With the liberalization of the "two-child" policy, the number of children in the family increases, and family members may give up using CRS due to the narrow space in the car [38]. Therefore, in addition to educating parents, educating other family members such as grandparents and relatives is also very important. In addition, car and safety seat manufacturers should consider the interior space layout of multi-child families, and how to further optimize the design of safety seats with wide applicability and portability, so that they can play a protective role in the limited car interior space.

The study also indicated that CRS use relevant knowledge, including children's riding mode cognition and CRS type cognition, was an important factor affecting parents' use of CRS, which is in line with the findings of earlier studies [39–41]. The likelihood and severity of a child's injury following a vehicle collision will increase if there is not an appropriate and effective restraint in the vehicle [42]. For instance, there will be significant safety risks for kids if parents sit in the back with kids in their arms and put the seat belts on them too early. Parents need to master solid knowledge to choose the right mode of transportation and the type of constraint for their children, rather than only rely on subjective judgment. Therefore, relevant departments must regularly educate parents of young children and widely disseminate pertinent information in order to decrease the incidence of riding-related injuries in kids.

Similar to the work by Li et al. [43], our study found that the use motivation of CRS, including the awareness of the serious injury caused by motor vehicle collisions to children and the parents' cognition of the use CRS benefits, are major influencing factors to promote the use of CRS. Currently, due to rapid development of the internet, information about children's injuries brought on by the improper administration of CRS can be rapidly disseminated online. Parents have a more intuitive understanding of the awareness and severity of children's riding injuries, so that they could take measures to prevent injury [44]. Additionally, the authority suggestion promotes the use of CRS, which is compatible with the research findings by Jin [45]. In Malaysia, a study on the factors influencing parents' decision to use CRS found that 42.7% of parents used CRS as a result of advice from medical professionals and nurses as well as recommendations from friends and family [46]. The possible reasons include: CRS involves a relatively professional field, it needs guidance from experts from different fields. Their guidance may be the basis and motivation to eliminate the obstacles to parents’ CRS using [47]. In addition, CRS research was just recently conducted in China, where CRS is less well known and understood by parents than those in other nations [48]. Therefore, parents in China would be strongly urged to utilize CRS for their children when they receive advise from specialists such as medical personnel and get recommendations from friends and family members nearby [45]. In the future, together with strengthening the education of parents' theoretical knowledge, improving parents' motivation must be particularly stressed.

The current study also shown that CRS was utilized more frequently if parents had greater installation skills. Parents of children in Shantou and Chaozhou kindergartens investigated by Yan et al. [49] received training in restraint systems and behavioral skills had the highest utilization rate and correct use rate of CRS, indicating that the combination of theoretical knowledge and practical operation could produce a better result than that only including knowledge component. This is consistent with the IMB's proposition [18]. Due to the professionalism and sophistication of CRS installation, the safety of children while riding would be gravely compromised if parents were unable to obtain dependable CRS installation skills [50]. Therefore, in addition to introducing information about CRS use, training on pertinent behavior skills of CRS installation, including the installation location and interface type, will be significant to encourage parents' use willingness and behavior of CRS in programs of popular science publicity and health education for parents in the future.

## Conclusion

In summary, the study results revealed that parents' cognitive, motivation and behavioral skills of CRS have a significant impact on increasing parents' use of CRS, which provides a direction and theoretical basis for designing more targeted programs to increase the use of CRS in the future. Apart from continuing to strengthen and disseminate relevant laws and regulations, government departments also need to provide financial assistance to parents to reduce the burden of using CRS. In addition, further study needs to focus on knowledge and skill training provided by hospitals, communities, and child care centers. In the meantime, healthcare professionals and experts in various fields should give full play to their guiding role, inspire parents to use CRS, and raise their awareness of safety precautions.

### Limitations

There are some limitations to this study. First, the questionnaire design did not consider the psychosocial factors related to the use of CRS and whether the children had experienced traffic injuries in the car. Second, the survey area is the central urban area of Qingdao. The parents had relatively higher education and better family economic conditions. Therefore, the extrapolation of the study findings in economically underdeveloped areas may be limited. In addition, Questionnaires are collected in a combination of online and offline methods. During the online surveys, if the survey respondents have questions, the investigators cannot reply in time; during the offline surveys, the research subjects may be more inclined to choose the good results because the investigators are on-site, which may lead to biased questionnaire results. Another weakness of the study is that behavioral skills were assessed on the basis of parental self-reports rather than observation. This can lead to bias. In the future, observation and interview methods need to be added to better supplement the research results.

## Supplementary Information

**Additional file 1.** General information questionnaire.

**Additional file 2.** IMB model questionnaire.

## Data Availability

The datasets used and/or analyzed during the current study are available from the corresponding author on reasonable request.

## Abbreviations

CRS: Child restraint system
IMB: Information-motivation-behavioral skills model
SPSS: Statistical package for social science
CI: Confidence intervals
