# Supplementary material for: Analysis of factors influencing the use of child restraint system by parents of children aged 0–6 years: an information, motivation, behavioral skills model-based cross-sectional study
Source: BMC Pediatr. 2023 Jan 2;23:2. doi: 10.1186/s12887-022-03827-9 (PMC9806879; doi:10.1186/s12887-022-03827-9)
Supplement: Supplementary file 1 — Additional file 1. General information questionnaire. [file 12887_2022_3827_MOESM1_ESM.docx]

**General Information Questionnaire**

**Demographic information**

1. Are you the child's father or mother

□ father □ mother

1. Your education level

□ Bachelor degree or above □ junior college

□ High school, technical secondary school or technical school

□ Junior high school and below

1. The average monthly income of a family (yuan)

□ ≤3000 □ 3001-6000 □ 6001-9000 □ >9000

1. Your family status

□ Only child □ Non only child

1. Your child's gender

□ boy □ girl

1. Child age group (years)

□ 0-3 □ 4-6

1. Children's Household registration

□ this city □ Other provinces and cities

1. Your housing ownership

□ Personally owned □ Rent a house

1. Have you participated in the training on child unintentional injury

□ Yes □ No

1. CRS usage frequency

□ High frequency use (CRS being used over 5 times in an average of 10 rides)

□ low frequency use (CRS being used between 1 - 5 times in an average of 10 rides)

□ Non-use (CRS is not used)
