# Supplementary material for: Analysis of factors influencing the use of child restraint system by parents of children aged 0–6 years: an information, motivation, behavioral skills model-based cross-sectional study
Source: BMC Pediatr. 2023 Jan 2;23:2. doi: 10.1186/s12887-022-03827-9 (PMC9806879; doi:10.1186/s12887-022-03827-9)
Supplement: Supplementary file 2 — Additional file 2. IMB model questionnaire. [file 12887_2022_3827_MOESM2_ESM.docx]

|  | **Dimension** | **Number** | **Item** |
| --- | --- | --- | --- |
| Knowledge | Riding mode cognition | 1 | The safety belt is designed according to the needs of adults |
|  |  | 2 | The safest way for children to ride is to be held by adults |
|  |  | 3 | Children sit in the back when riding, and it is safe without any restraint |
|  |  | 4 | Children under 4 years old must use child restraint system when riding |
|  | Type cognition | 5 | Heightening the seat is an important step between the car seat and the safety belt |
|  |  | 6 | Children aged 3 should use forward facing child restraint system |
|  |  | 7 | Many children over the age of 8 should still use booster seats |
| Motivation | CRS application motivation | 8 | I believe that unintentional injuries to children will cause a heavy burden to children and families |
|  |  | 9 | I think the pamphlet or foldout on children's riding safety education is helpful to me |
|  |  | 10 | I think what my friends, colleagues or neighbors say about the use of child safety seats will affect me |
|  |  | 11 | I think what the medical staff said about the use of child restraint system will affect me |
|  |  | 12 | I think the use of child restraint system will reduce my worries about children travelling by car |
| Behavioral skills | Self-efficacy | 13 | I know how and can use the correct restraint for my child to prevent injury in a car accident |
|  |  | 14 | I have the skills and knowledge required to use the correct safety restraints to reduce the chance of my child being injured in a car accident |
|  |  | 15 | I can use the recommended restraints for my child to prevent him / her from being injured in a car accident |
|  | Installation skills | 16 | The child restraint system is divided into ISOFIX, latch and safety belt installation |
|  |  | 17 | As long as my child is constrained in some way, I think the type of constraint or how to install it is not so important |
|  |  | 18 | When the child restraint system is tightly installed in the vehicle, the seat movement range shall be limited to 3 cm |

**IMB model questionnaire**
